# Enhancing histology education: Iranian medical students’ experience with eHistoLab web-application virtual microscopy

**DOI:** 10.1186/s12909-026-09829-w

**Published:** 2026-07-01

**Authors:** Mahnaz Poorhassan, Manijeh Hooshmandja, Zahra Karimian, Elham Seifali

**Affiliations:** 1 Department of Anatomical Sciences and Cognitive Neuroscience, TMS.C, Islamic Azad University, Tehran, Iran; 2 Department of e-Learning in Medical Sciences, Virtual School, Center of Excellence in e-Learning, Shiraz University of Medical Sciences, Shiraz, Iran

**Keywords:** Histology education, Anatomy education, Virtual microscopy, Online learning, eHistoLab

## Abstract

**Background:**

Histology education relies on the visual interpretation of microscopic structures and requires repeated practice for effective learning. Advances in educational technology, particularly virtual microscopy, have created new opportunities for improving histology education. During the COVID-19 pandemic, online learning approaches became essential alternatives to traditional laboratory-based teaching. This study aimed to compare the effects of the eHistoLab web application and conventional e-lecturing on students’ learning outcomes and perceptions in histology education among medical sciences students in Iran.

**Methods:**

This quasi-experimental study used a two-group pre-test/post-test design. A total of 100 medical, dentistry, and pharmacy students enrolled in a histology course at Smart University of Medical Sciences (SMUMS), Iran, during the summer semester of 2023 participated in the study. Participants were randomly assigned into two groups. One group received instruction through e-lecturing, while the other group was taught using the eHistoLab web application, a bilingual virtual microscopy platform providing access to high-resolution histological slides, image magnification, search functions, and interactive communication with instructors. Learning outcomes were assessed using pre- and post-tests, and data were analyzed using paired and independent t-tests in SPSS version 24.

**Result:**

The findings revealed that 50% of students preferred virtual microscopy alone for learning histology, while 30% preferred a combination of methods, and 20% preferred e-lecturing alone. Regarding educational resources, 58% of students preferred the eHistoLab application, whereas 26% preferred textbooks and 16% preferred recorded e-lectures. Meantime, 62% of students preferred the eHistoLab tool in terms of motivation for learning histology, whereas 30% preferred the combined approach and 8% favored recorded e-lectures. Moreover, the results indicated that the post-test scores in the group taught with the eHistoLab web application were significantly higher than those in the e-lecturing group (*P* < 0.001).

**Conclusion:**

The findings of this study suggest that the eHistoLab web application improved students’ learning outcomes and was positively perceived as a learning tool in histology education. Virtual microscopy may therefore serve as a useful complementary approach to lecture-based histology teaching.

## Introduction

Although medical students will ultimately work in a clinical environment, they need to be familiar with the basic medical sciences and acquire the basics before entering the clinical phase. Histology, formally known in anatomical language as microscopic anatomy, constitutes one of the most important courses in basic medical sciences. It is included in the curriculum of this discipline [1] and is a challenging subject necessitating practice and repetition for effective learning [2].

Macroscopic and microscopic anatomy is a fundamental requirement for most clinical specialties due to the necessity of safety and efficiency. However, much of histology nomenclature continues to remain strange to fresh students due to the complexity of the discipline. The complexity of histological terminology may increase cognitive load among novice learners, thereby hindering their understanding and interpretation of histological concepts [3]. Competitive pressure on curricula has forced numerous medical schools to scale back on the time allocated to normal histology [4].

Histology education is often resource-intensive, particularly in institutions with limited or aging microscope equipment and inadequate histological slide collections [5]. A growing number of student enrolments requires laboratory course duplication. Practical classes are often staff-intensive, thereby requiring teaching hours to increase [6]. Reliance on inadequate resources may adversely affect the learning experience of students, resulting in a superficial understanding of histology. Students are used to memorizing visual information without fully grasping the underlying concepts of microanatomy [5].

To address these challenges, medical educators continuously seek innovative teaching strategies to improve student engagement and learning outcomes. Enhancing student motivation and addressing negative perceptions of histology among medical, dental, and nursing students remains an important educational goal [7].

In recent times, technological developments in educational technologies such as Virtual Reality (VR) and Augmented Reality (AR), 3D visualization software, e-learning platforms, and digital microscopy have greatly impacted health professions education [8, 9]. Among these technologies, Virtual Microscopy (VM) has emerged as one of the most important in histology education.VM can be defined as the use of advanced scanning methods to digitally represent histological slides. This allows one to interact with and zoom into the histological image by computer software without having to use an optical microscope. VM enables remote access to histological specimens and supports self-directed, flexible learning environments in medical education [10].

A number of studies have been published showing how VM can be used as a replacement or complement to conventional microscopy in teaching histology. Benefits of VM include increased accessibility and learning motivation, as well as flexibility in limited-resource environments [11–13]. VM has been used in histology education for more than two decades, and numerous studies have compared its effectiveness with conventional light microscopy and didactic teaching approaches, generally reporting comparable or improved learning outcomes [14, 15].

However, despite this extensive literature, the implementation and pedagogical integration of VM systems vary widely across institutions. Moreover, studies investigating the efficiency of VM programs created locally and specific to each curriculum, which can operate bilingually, are scarce. To meet this research requirement, the eHistoLab application software was designed as a virtual microscopy platform with interactive and bilingual (Persian and English) capabilities. Unlike other VMs, where users mostly have a role only in browsing through a set of pre-downloaded images, eHistoLab includes advanced features like high-resolution images, navigation, searching, communication, and integration with Learning Management System (LMS). Therefore, the present study aimed to compare the effectiveness of the eHistoLab web application with conventional e-lecturing in histology education among medical sciences students and to evaluate students’ perceptions of this learning approach.

## Methods

### Study design

This study was carried out using a quasi-experimental two-group design based on pre-test and post-test knowledge assessment. Two groups of students of medicine, dentistry, and pharmacy who had taken the histology course in the summer semester of 2023 at Smart University of Medical Sciences (SMUMS), Iran, participated in this research. Each group received instructions in a different method, either e-Lecturing or eHistoLab web application combined with recorded e-Lecturing sessions.

### Participants and sampling

A total of 250 s-year medical, dental, and pharmacy students were enrolled in the practical histology course during the summer semester of 2023 (15 July 2023–15 September 2023). These students were distributed across several instructional classes taught by different instructors. To minimize the potential confounding effects of variations in teaching style, instructional delivery, and classroom management, only one class consisting of 100 students who received identical educational content from the same instructor under similar learning conditions was selected for this study.

The selected students were randomly assigned to either the conventional online histology group or the eHistoLab web application group using a simple randomization (lottery) method. Sample size estimation based on comparison of means between two independent groups, with a confidence level of 95% and statistical power of 80%, indicated that at least 40 participants were required in each group. To compensate for possible attrition, 50 students were allocated to each group. Accordingly, the inclusion of 100 students from the selected class exceeded the minimum required sample size and provided sufficient statistical power to detect meaningful differences between the study groups.

### Tools/ instrument

#### Demographic characteristics

The data on the student’s gender and their field of study were collected at the start of the questionnaire.

#### Q1. Perception assessment

To assess the students’ perceptions of the eHistoLab web application method, a customized questionnaire was designed based on the questionnaire developed by Tauber et al. [16]. The questionnaire consisted of 3 main sections with a total of 9 items: *Preferred tools* (3 items), *Preferred Educational Resources* (3 items), and *Motivation*. Students were allowed to select only one answer for each question.

#### Validity/ reliability

To determine the face validity of the questionnaire, it was initially presented to 10 students. Then, based on their feedback, 3 questions required grammatical changes. Additionally, one item was modified to align with the educational method being implemented. The instrument was reported to have a Content Validity Index (CVI) of approximately 0.86 in Tauber et al.‘s [16] research. However, the opinions of 4 histology and medical education experts were sought to re-evaluate the questionnaire. In previous research, the reliability of the questionnaire was reported to be 0.87.

#### Q2. Knowledge assessment

To assess students’ learning outcomes (knowledge), a final examination consisting of 50 multiple-choice questions (each with one correct answer) was administered. All questions were equally weighted, and no negative marking was applied. The exam scores were converted to a scale of 0 to 10. The passing score was set at 5. A score between 5 and 7 was considered satisfactory, 7 to 8.5 good, and 8.5 to 10 excellent.

In addition, to evaluate knowledge acquisition, a pre-test was administered to both groups (the eHistoLab web application group and the e-lecturing group) before the intervention, and a post-test was conducted after completion of the course.

It deserves mentioned that the questions for this test were selected from the university’s standardized and repeated question bank, with similar difficulty and discrimination indices. Additionally, the equivalence or matching of the questions in the pre-test and post-test was confirmed by the judgment of two collaborating instructors of histology.

#### Blinding

Participants attended the course virtually and were unaware of the existence of the alternative study group. The same pre-test and post-test assessments were administered to both groups. Data analysis was performed by an independent statistician who was blinded to group allocation.

#### Educational content

The histology reference for professional doctorate students is Junqueira’s Basic Histology, according to the curricula decided by the Iranian Ministry of Health, Treatment and Medical Training (reference). It is presented in ten educational sessions, each lasting approximately 1 ½ hours. The educational content was identical for both groups and all students had access to the Learning Management System (LMS). The students had registered through the central education system. The course consisted of 10 practical sessions based on systemic histology: nervous, digestive, respiratory, male reproductive, female reproductive, urinary, circulatory, special senses (eye and ear), and immune systems.

It should be noted that the content of the e-lecturing sessions (learning objectives, teaching materials, and instructor) was identical for both groups; the only difference was the mode of delivery.

#### Educational Intervention

The 100 sampled participants were randomly divided into two groups at the beginning.

#### a. Conventional online histology (e-Lecturing) intervention

This group received 10 educational sessions through virtual classrooms conducted by the course instructor using the university’s routine online teaching method via Adobe Connect. Instruction was delivered using PowerPoint presentations. During each session, students had the opportunity to ask questions through the chat function, and responses were provided at the end of the session.

The recorded sessions were uploaded to the Learning Management System (LMS), enabling students to review the materials and reinforce their learning outside the scheduled class time.

#### b. eHistoLab web application intervention

The eHistoLab group received instruction through a blended learning approach consisting of the eHistoLab web application and access to instructor-recorded lectures uploaded to the Learning Management System (LMS). The recorded lectures were identical to those provided to the conventional online histology group and enabled students to review the course content throughout the study period.

Access to the eHistoLab web application was restricted through individual usernames and passwords. User accounts were created exclusively for students assigned to the intervention group, and access to the application was not available to students in the comparison group. This measure was implemented to prevent cross-group contamination and ensure that only participants in the intervention group could use the eHistoLab platform during the study period.

Through the LMS, students in the eHistoLab group also had access to the same recorded lectures, assignments, quizzes, and educational materials provided to the comparison group. Therefore, the primary difference between the two groups was the use of the eHistoLab web application as a practical histology learning tool.

With the fact that students voluntarily enrolled in the virtual histology course, all of them had access to smartphones. During the educational intervention phase, the software was provided to the selected research participants through Firebase App Distribution via a private link, which was sent to the email addresses of the selected students. However, after the completion of the study, public access to the application was enabled and it became available through Google Play (Software link).

### Key features of the application

The eHistoLab application is an Iranian-developed mobile learning platform designed and implemented by *Shiraz Mobile Anatomical Lab Pioneers* at Shiraz University of Medical Sciences (SUMS) to support histology education. The scientific content, laboratory images, and educational resources were developed and curated by faculty members of the Departments of Anatomy and Histology. The application employs virtual microscopy technology and incorporates a library of approximately 130 high-quality histological slides that were digitized through whole-slide scanning and converted into high-resolution virtual images. These slides are accessible through an integrated virtual microscope viewer, enabling users to navigate tissue sections and examine structures at magnifications of up to ×400. Designed primarily for smartphone-based learning, eHistoLab provides students with convenient access to histology learning materials anytime and anywhere. Following evaluation and approval by the School of Medicine of Shiraz University of Medical Sciences, the application was published on the Google Play Store, making its educational resources available to students and educators worldwide.

**Fig. 1:**
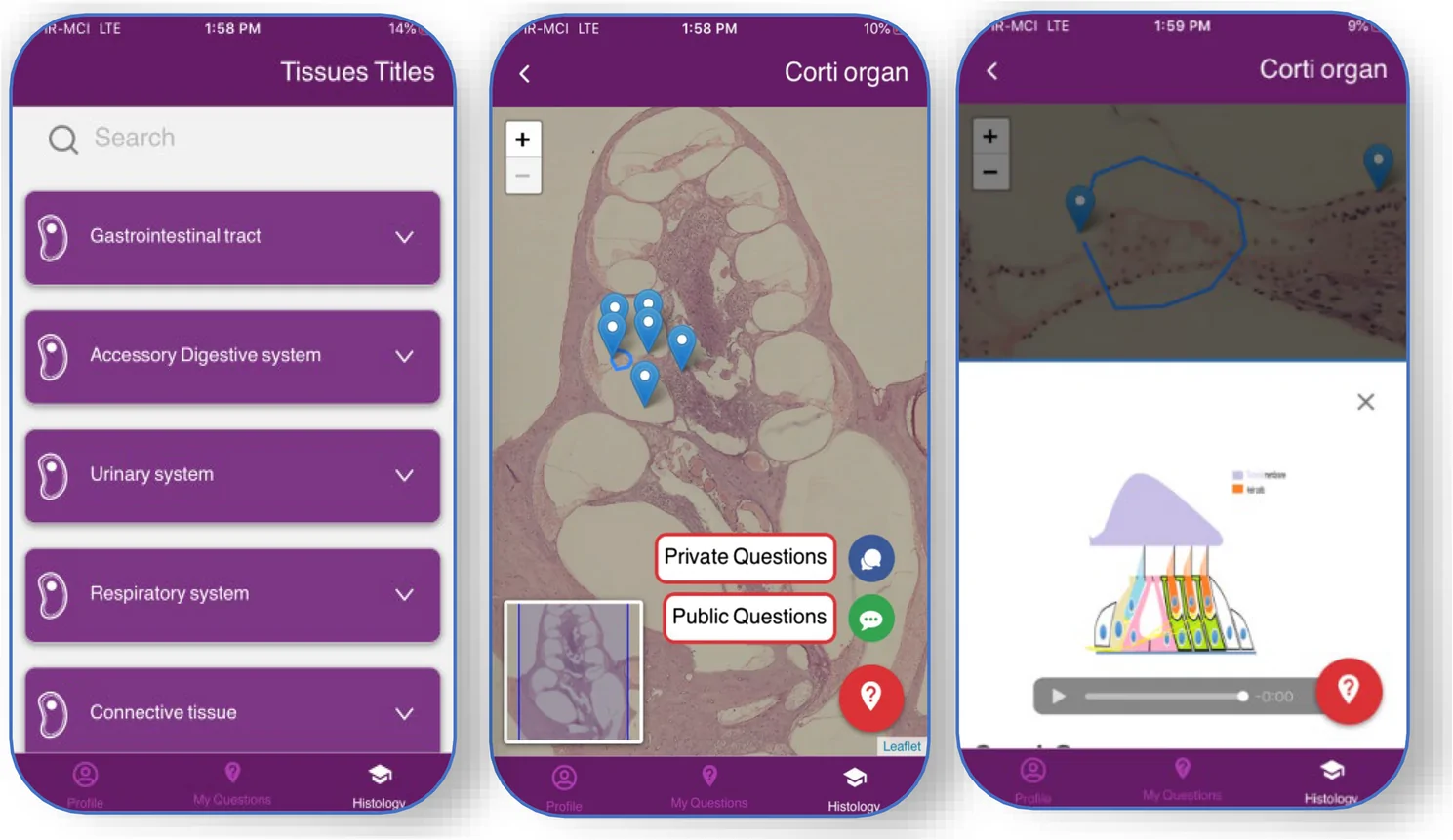
A view of the eHistoLab Web Application

The main features of the application are presented in Fig. 1 and include:

- ○ High-quality schematic images for a better understanding of the content.
- ○ The ability to exchange with professors in public and private forums.
- ○ The option to switch to Persian or English.
- ○ The ability to transfer most visited slides to the favorites section.
- ○ Search functionality for finding tissue structures more quickly.
- ○ The possibility to exchange with the instructor during online training.

#### Assignments and quizzes

Both groups had access to the university’s Learning Management System (LMS) for course administration, assignment submission, quizzes, and distribution of educational materials. The assignments, quizzes, and learning resources available through the LMS were identical for both groups. Therefore, the only difference between the groups was the practical histology learning method used during the intervention period.

**Fig. 2:**
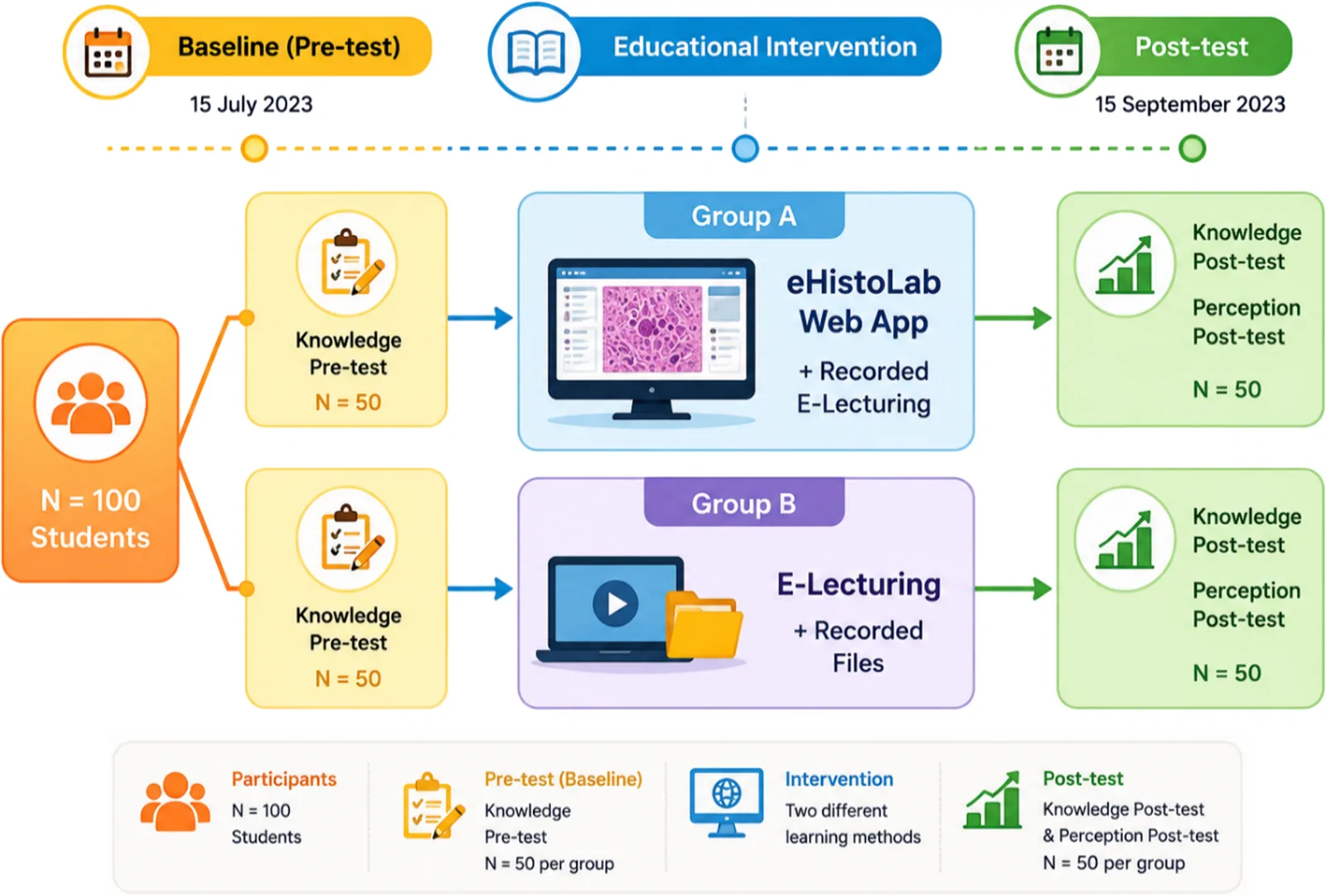
Schematic diagram of the research design and intervention

A schematic representation of the research design is shown in Fig. 2.

### Data collection

To assess the students’ perceptions of their learning experience with eHistoLab (main intervention group), an electronic questionnaire, designed by Google Forms, was used. The questionnaire link was emailed to the students after the completion of the course. The pre-test and post-test to assess learning (knowledge) were also designed as online electronic tests with multiple-choice questions (with one correct answer) and no negative scores. Both groups of students participated simultaneously in the pre-test and post-test within a specified timeframe.

All instructional sessions for both groups were conducted in an online format via the university Learning Management System and virtual classroom platform. Students participated remotely from their own residences, and all sessions were supervised by the course instructor to ensure adherence to the study protocol and appropriate engagement.

### Data analysis

To determine the students’ perceptions, a descriptive method (frequency and percentage) was employed. For the purpose of comparing the pre-test and post-test mean scores within each group, the paired t-test was used, while comparing the pre-test and post-test between the groups was done by the independent t-test. The data was analyzed by the SPSS version 24 software.

## Results

### Demographic characteristics

All 100 students who took part in the pre-test also participated in the post-test, and none of them dropped out. Additionally, 50 students from the eHistoLab group responded to the questions on their perception. Table 1 shows the detailed demographic information of the participants from both groups. The demographic characteristics of the participants were identical in both groups in terms of the distribution of gender and field of study. (Table 1).

**Table 1:** Demographic characteristics of participants

Variables		eHistoLab web application		e-Lecturing	
		Frequency	(%)	Frequency	(%)
Gender	• Male	23	46.0	19	38.0
	• Female	27	54.0	31	62.0
	• Total	50	100.0	50	100.0
Field of Study	• Medicine	25	50.0	23	46.0
	• Dentistry	13	26.0	14	28.0
	• Pharmacy	12	24.0	13	26.0
	• Total	50	100.0	50	100.0

### Findings from perception measurement

Students generally reported positive perceptions toward the use of virtual microscopy in histology education. Most participants considered virtual microscopy, either alone or in combination with e-lecturing, to be more effective than recorded e-lecturing alone for learning histology. Virtual microscopy was the most frequently preferred educational resource and was associated with higher reported motivation for learning histology. Detailed findings are presented in Table 2.

**Table 2:** Students’ preferences regarding features of educational tools

Questions	Medicine (*N* = 25)	Dentistry (*N* = 13)	Pharmacy (*N* = 12)	Total (*N* = 50)
Which tool do you consider to be the most effective in learning histology?				
• Only virtual microscopy	%22(11)	%12(6)	%16(8)	50% (25)
• Only recorded e-Lecturing	%14(7)	%2(1)	%4(2)	20% (10)
• Combination of both	%14(7)	%12(6)	%4(2)	30% (15)
Which sources do you prefer in learning histology?				
• Virtual microscopy	34% (17)	%14(7)	10% (5)	58% (24)
• Recorded e-Lecturing	8% (4)	%4(2)	%4(2)	16% (8)
• Textbook	8% (4)	8% (4)	10% (5)	26% (13)
Which tool motivates you to learning histology?				
• Only virtual microscopy	36% (18)	14% (7)	12% (6)	62% (31)
• Only recorded e-Lecturing	%4(2)	%2(1)	%2(1)	8% (4)
• Combination of both	10% (5)	10% (5)	10% (5)	30% (15)

**Fig. 3:**
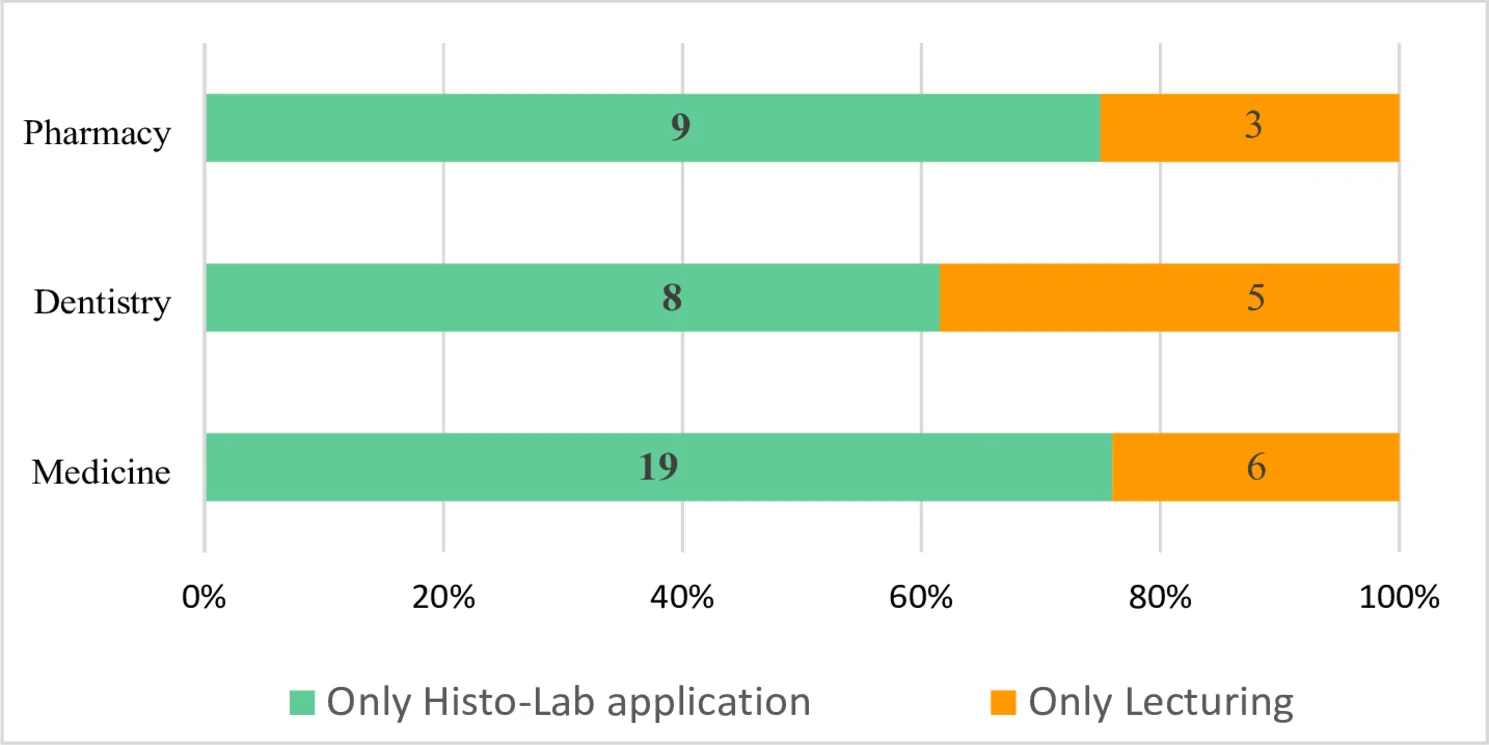
Preferred tools from students’ perception in only-one option situation

In the fourth question, we asked students: “If you could only choose between two options, i.e. eHistoLab and electronic lecture, which one would you prefer?” The majority preferred the eHistoLab option (Fig. 3).

### Findings from knowledge measurement

A paired t-test was used to compare pre-test and post-test knowledge scores within each group. The results indicated that post-test scores were significantly higher than pre-test scores in both the e-Lecturing and eHistoLab groups (*P* < 0.001), demonstrating that both instructional methods improved students’ knowledge. Independent t-test analysis showed no significant difference between the groups in pre-test scores (*P* = 0.62). However, post-test scores in the eHistoLab web application group were significantly higher than those in the e-Lecturing group (*P* < 0.001). The effect size for the between-group post-test comparison was large (Cohen’s d = 1.31). (Table 3).

**Table 3:** Comparison of students’ knowledge between two intervention groups

Knowledge of students						
Groups	N	t	Average Score		P-value	Cohen’s d
			Pre-test	Post-test		
E-Lecturing + recorded files	50	-35.64	1.891 ± 0.90	6.31 ± 1.02	< 0.001	1.31
eHistoLab + recorded e-Lecturing	50	-14.77	1.980 ± 0.91	7.65 ± 1.02	< 0.001	
Between-group comparison	100	---	*P* = 0.62 t = 0.496	*P* < 0.001 t = 6.36	---	

Comparison of post-test scores based on gender showed that female students achieved slightly higher scores than male students; however, the difference was not statistically significant (*P* = 0.502).

## Discussion

The findings of the present study suggest that students perceived virtual microscopy as a valuable and engaging approach for learning histology, consistent with previous studies reporting positive student perceptions toward virtual microscopy in medical education [17–24]. Participants generally favored the eHistoLab web application either alone or in combination with recorded lectures, indicating that digital microscopy may enhance students’ learning experience and motivation in histology education.

Student motivation represents a key factor in the learning process [25, 26]. Motivated students have been shown to be more active and participative in class, as well as more proactive in knowledge and information acquisition. In line with this, previous studies have also reported that students tend to prefer digital learning methods over conventional approaches, with student satisfaction being considered an important indicator of learning outcomes [27].

Previous research has consistently reported positive perceptions toward the use of virtual microscopy in histology education. The accessibility of slide images outside the classroom [17–20], the possibility of annotating slides, and the availability of abundant digital resources have made this method more efficient and feasible for learning [15, 22–24]. Studies investigating the use of virtual microscopy in practical training sessions have also indicated improved ease of learning histology among students [20].

In the present study, regarding preferred educational resources, 58% of students considered the eHistoLab application alone to be sufficient, 26% emphasized the use of textbooks, and 16% considered recorded e-lectures to be appropriate.

Students also reported positive perceptions regarding the use of the eHistoLab application as a learning resource. Virtual microscopy offers numerous advantages compared with traditional methods, including easier access to learning materials inside and outside the laboratory, overcoming limitations related to space, equipment availability, and scarcity of specimens [28, 29].

Virtual microscopy allows students access to content anytime and anywhere while also offering opportunities for educator-guided interactivity and learning [30]. From this perspective, it appears particularly suitable for histology education because it supports image-based recognition and interpretation of microscopic structures.

A distinguishing feature of the eHistoLab web application is its integration of virtual microscopy with several educational support tools within a single platform. Unlike many publicly available virtual microscopy resources, such as Histology Guide and similar web-based repositories, which primarily focus on slide visualization and self-study, eHistoLab combines high-resolution histological slides with bilingual (Persian/English) support, instructor–student communication through public and private discussion forums, search functionality for tissue structures, bookmarking features, and integration with the institutional learning management system. These features were specifically designed to address the educational needs of Iranian medical sciences students and to facilitate both independent and guided learning. The availability of Persian-language content may be particularly valuable for novice learners who are still developing proficiency in English medical terminology, as part of a structured educational intervention within the medical curriculum.

The improved learning outcomes observed in the present study may be explained by several educational mechanisms associated with virtual microscopy. It provides repeated exposure to histological structures, supports active visual exploration, and facilitates image-based pattern recognition, which are essential components of histology learning [15]. In addition, the ability to navigate, zoom, and repeatedly review digital slides may help reduce cognitive load and improve visual-spatial processing compared with conventional static learning approaches [15–31]. These features may enhance students’ conceptual understanding and retention of microscopic anatomy [32].

Accordingly, a study compared a lecture-based group with a group trained using virtual microscopy and found that students trained with virtual microscopy showed improved short-term learning and retention. However, no significant difference was observed between the groups in the long term [33].

According to the findings of a study, students used a combination of VM and textbooks, with most of them using electronic resources regularly [34].These findings support the complementary role of virtual microscopy alongside conventional educational resources rather than as a complete replacement for them.

Meantime, despite the numerous advantages of various digital technologies, it was noted that the introduction of Digital Information and Communication Technologies may yield such negative impacts as decreased attendance and procrastination, in addition to a lack of student attendance in practical lessons, based on the assumption of the possibility of learning histology without teacher guidance [35].

Moreover, technology by itself cannot improve the learning outcome, suggesting the necessity of concurrent implementation of external frameworks of accountability to obtain a better output [36]. Lectures and textbooks mainly support theoretical knowledge acquisition, whereas virtual microscopy primarily enhances visual recognition and interpretation skills in histology. Therefore, these approaches should be considered complementary rather than competing components of the histology learning process.

In the present study, both instructional methods improved students’ knowledge scores. However, students in the eHistoLab group achieved significantly higher post-test scores compared with the e-Lecturing group, suggesting that virtual microscopy may provide additional benefits in histology learning when combined with lecture-based instruction.

Similar findings have been reported in previous studies evaluating virtual microscopy in histology and pathology education. Earlier research demonstrated that virtual microscopy improves students’ learning experiences, accessibility to histological materials, and academic performance compared with traditional microscopy methods. Studies by Wu and Chiang [37], Kumar et al. [38], and Schoenfeld-Tacher and McConnell [39] reported greater student satisfaction, improved learning outcomes, and enhanced interaction in virtual or online histology learning environments. Similarly, Barbeau et al. [40] found that online digital histology education achieved learning outcomes comparable to traditional and blended approaches. Furthermore, a systematic review and meta-analysis by Maity et al. [28] concluded that virtual microscopy is generally associated with better academic performance, ease of use, and improved collaboration among students. Consistent with these findings, the present study demonstrated that the eHistoLab web application enhanced students’ learning outcomes and was positively perceived as an effective educational tool in histology education.

These findings are supported by foundational studies on virtual microscopy in medical education. Paulsen et al. highlighted its value in improving accessibility, flexibility, and student-centered learning, as well as the importance of appropriate curricular integration [41]. Similarly, Darici et al. showed that fully digital histology teaching during the COVID-19 pandemic could maintain student engagement and learning outcomes despite the limitations of remote education [24].

Recent studies have emphasized the importance of interactive and visually enriched teaching approaches. Video-assisted, 3D, and narrative-based methods have been shown to improve engagement, conceptual understanding, and academic performance compared with traditional approaches [42–44], consistent with the findings of the present study.

With the expansion of virtual learning platforms, VM has been increasingly adopted in undergraduate and preclinical education [45], and its use has grown significantly in recent years alongside the broader shift toward digital learning [46–50]. Clinical evidence also supports its diagnostic reliability, showing non-inferiority to conventional microscopy in histopathology [51, 52]. Despite these advantages, conventional approaches such as microscopic examination and gross specimen dissection remain essential for developing practical skills and understanding tissue morphology. Accordingly, a blended approach combining digital and traditional methods is recommended [53]. Digital technologies are also increasingly applied in histopathology across various fields, including dermatology, neurology, oncology, and gastrointestinal and hematological pathology [54, 55].

Beyond technical applications, virtual microscopy may contribute to competency-based medical education by supporting the development of core competencies such as medical knowledge, professionalism, and lifelong learning within modern medical curricula [22, 56–58].

The implications of the present findings may extend beyond the local educational context in which the study was conducted. Although Histo-Lab was developed for Iranian medical sciences students, the educational principles underlying the platform—including flexible access to virtual slides, repeated exposure to histological structures, learner-centered interaction, and integration with existing learning management systems—are applicable to histology education in a wide range of settings. These findings may be particularly relevant for institutions seeking cost-effective strategies to supplement laboratory-based teaching, expand access to microscopy resources, or support blended and online learning environments.

### Suggestions and limitations

There are some limitations of the present study that should be taken into account when interpreting the findings. First, the study was conducted at one institution, so the generalizability of the results to other educational settings is limited. Second, the eHistoLab group was exposed to a blended learning environment including virtual microscopy, recorded lectures, and an interactive web-based platform, whereas the comparison group received synchronous online instruction with direct instructor interaction. As a result, the interpretation of these results is limited to this specific instructional design. Furthermore, the evaluation was limited to short-term learning outcomes as assessed immediately following the intervention, while long-term retention of knowledge was not assessed. Any prior experiences students may have had with digital technologies and the online-based educational software used in the virtual microscopy course were not assessed; this could have negatively impacted the students’ participation, engagement and learning outcomes. Furthermore, learning outcomes were primarily assessed using multiple-choice examinations or via student self-reporting, and the students’ actual practical skills regarding the use of a microscope, as well as their ability to interpret an observed specimen immediately after observation, were not assessed. Differences in internet availability and/or the students’ level of readiness with digital technologies may also have impacted the level of participation or effectiveness of the student learning experience. longitudinal investigations of the long-term educational efficacy of VM within the study of histology are warranted.

## Conclusion

The findings of the present study demonstrated that the eHistoLab web application improved students’ learning outcomes and was positively perceived as an effective and engaging educational tool in histology education. Students in the eHistoLab group achieved significantly higher post-test scores compared with the e-lecturing group, suggesting that virtual microscopy may enhance histology learning when combined with lecture-based instruction. The eHistoLab application may facilitate students’ understanding and interpretation of histological structures by providing accessible, interactive, and image-based learning opportunities. Therefore, integrating virtual microscopy with conventional teaching approaches may enhance the effectiveness of histology education and enrich students’ learning experiences.

Although the study was conducted within a specific educational context, the educational principles underlying the eHistoLab platform—including flexible access to digital slides, learner-centered interaction, and opportunities for repeated exposure to histological structures—may be applicable to other medical education settings seeking to integrate virtual microscopy into blended or online histology curricula.

## Data Availability

The authors confirm that the data supporting this study’s findings are available in the article and its supplementary materials.

## Abbreviations

VM: Virtual Microscopy
